# Author Correction: Attenuation of RNA polymerase II pausing mitigates BRCA1-associated R-loop accumulation and tumorigenesis

**DOI:** 10.1038/ncomms16211

**Published:** 2018-03-30

**Authors:** Xiaowen Zhang, Huai-Chin Chiang, Yao Wang, Chi Zhang, Sabrina Smith, Xiayan Zhao, Sreejith J. Nair, Joel Michalek, Ismail Jatoi, Meeghan Lautner, Boyce Oliver, Howard Wang, Anna Petit, Teresa Soler, Joan Brunet, Francesca Mateo, Miguel Angel Pujana, Elizabeth Poggi, Krysta Chaldekas, Claudine Isaacs, Beth N. Peshkin, Oscar Ochoa, Frederic Chedin, Constantine Theoharis, Lu-Zhe Sun, Tyler J. Curiel, Richard Elledge, Victor X. Jin, Yanfen Hu, Rong Li

Nature Communications 8: Article number: 15908; DOI: 10.1038/ncomms15908 (2017); Published 06 26 2017; Updated 03 30 2018

The original version of this Article omitted the following from the Acknowledgements:

‘The work was also supported by a grant to Y.H. from the Cancer Prevention Research Institute of Texas CPRIT RP170126.’

This has been corrected in both the PDF and HTML versions of the Article.

