# Supplementary material for: Attenuation of RNA polymerase II pausing mitigates BRCA1-associated R-loop accumulation and tumorigenesis
Source: Nat Commun. 2017 Jun 26;8:15908. doi: 10.1038/ncomms15908 (PMC5490191; doi:10.1038/ncomms15908)
Supplement: Supplementary Information [file ncomms15908-s1.pdf]

Type of file: PDF
Size of file: 0 KB
Title of file for HTML: Supplementary Information
Description: Supplementary Figures and Supplementary Tables

Type of file: XLSX
Size of file: 0 KB
Title of file for HTML: Supplementary Data 1
Description: Overlaps between genes with BRCA1 mutation-associated R-loops and previously defined luminal signature genes.

Type of file: XLSX
Size of file: 0 KB
Title of file for HTML: Supplementary Data 2
Description: Differential expression analysis of microarrays in mouse mammary epithelia.

Type of file: PDF
Size of file: 0 KB
Title of file for HTML: Peer Review File
Description:

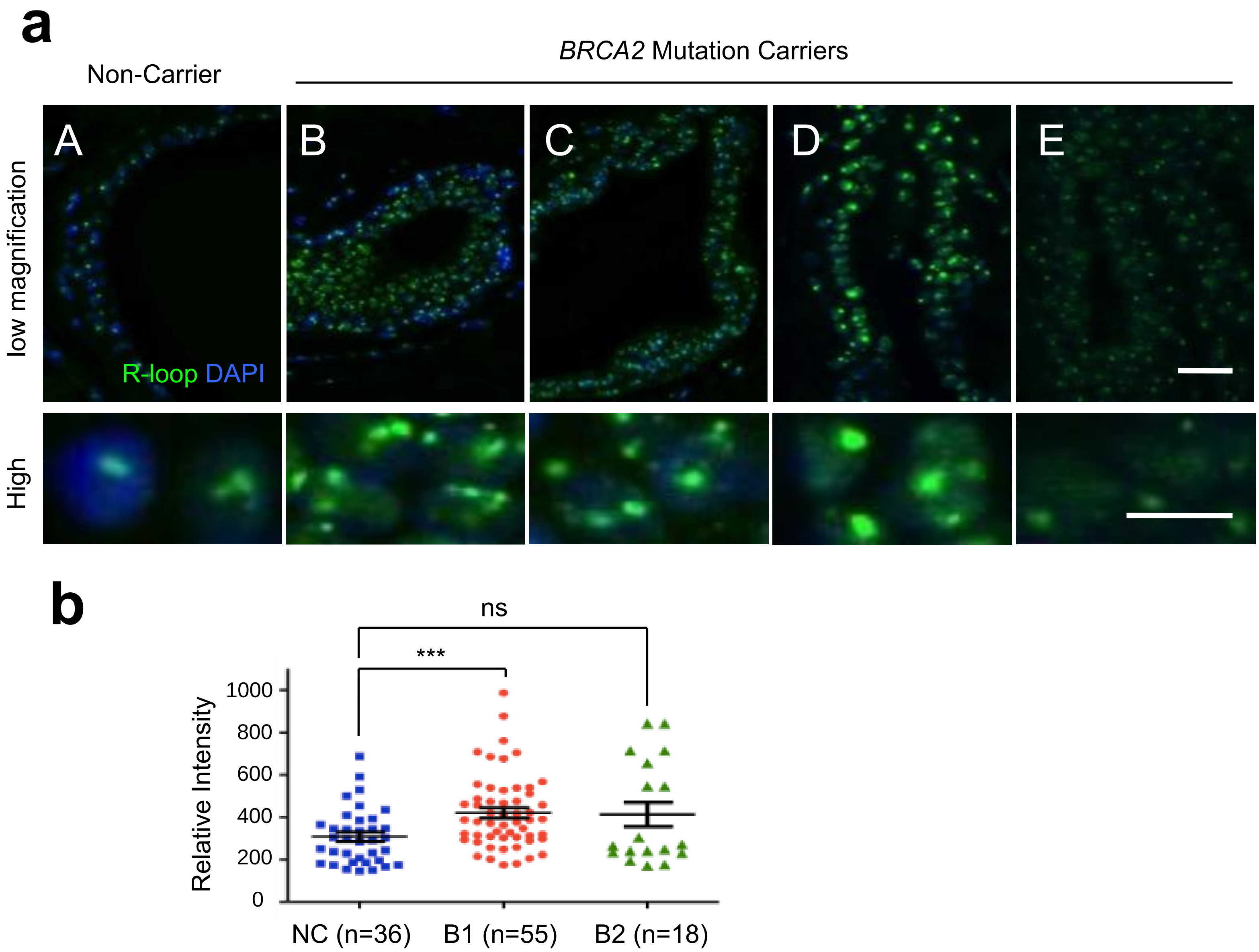

Supplementary Figure 1. *BRCA2* mutation-associated R-loop in cancer-free breast tissue. (**a**) Low and high magnification images of R-loop staining in samples from non-carriers and *BRCA2* mutation carriers. Scale bar: 20 μm (top) and 5 μm (bottom). (**b**) Quantitation of relative R-loop intensity in the non-carrier (NC) group (n=36), *BRCA1* (B1) mutation carrier group (n=55) and *BRCA2* (B2) mutation carrier group (n=18). *** $P < 0.001$ by two-tailed *t*-test. Error bars represent s.e.m. ns: not significant.

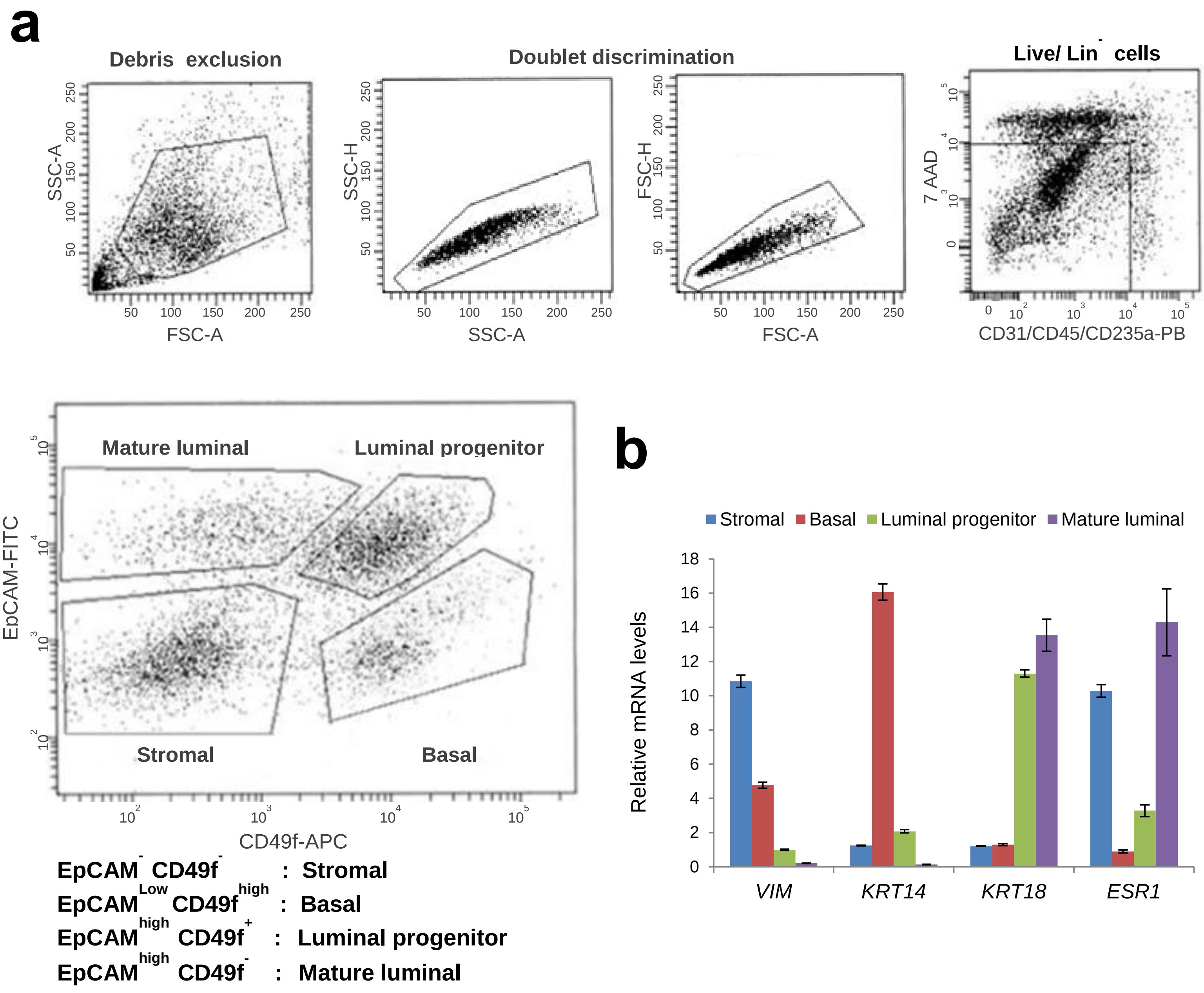

Supplementary Figure 2. Sorting of primary human breast epithelial and stromal cells. (**a**) Representative flow cytometry results indicating the typical gating for debris exclusion, doublet discrimination, lineage-negative/live cells selection, and separation of stromal, basal, luminal progenitor, and mature luminal cells. (**b**) Validation of cell sorting efficiency by RT-PCR of known stromal (*VIM*), basal (*KRT14*), luminal (*KRT18*) and mature luminal (*KRT18* and *ESR1*) markers. *ACTB* was used for normalization.

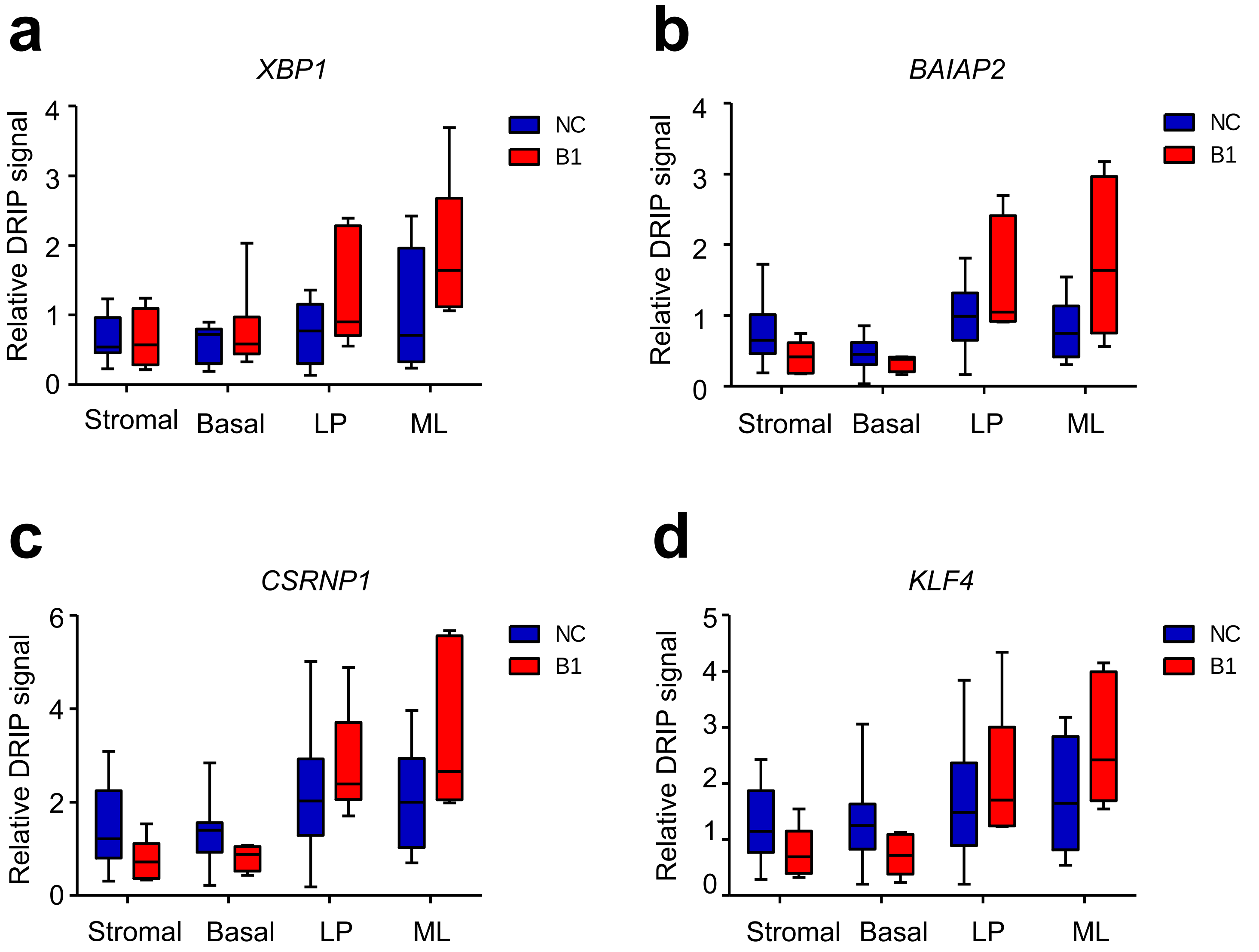

Supplementary Figure 3. Confirmation of *BRCA1* mutation-associated R-loop enrichment by PCR. (**a-d**) Validation of DRIP-seq by RT-PCR of genes with *BRCA1* mutation-associated TSS R-loops. Box and whisker plot showing relative DRIP signal at *XBP1* (a), *BAIAP2* (b), *CSRNP1* (c) and *KLF4* (d). The number of cancer-free breast tissues used: NC (n=9), B1 (n=6).

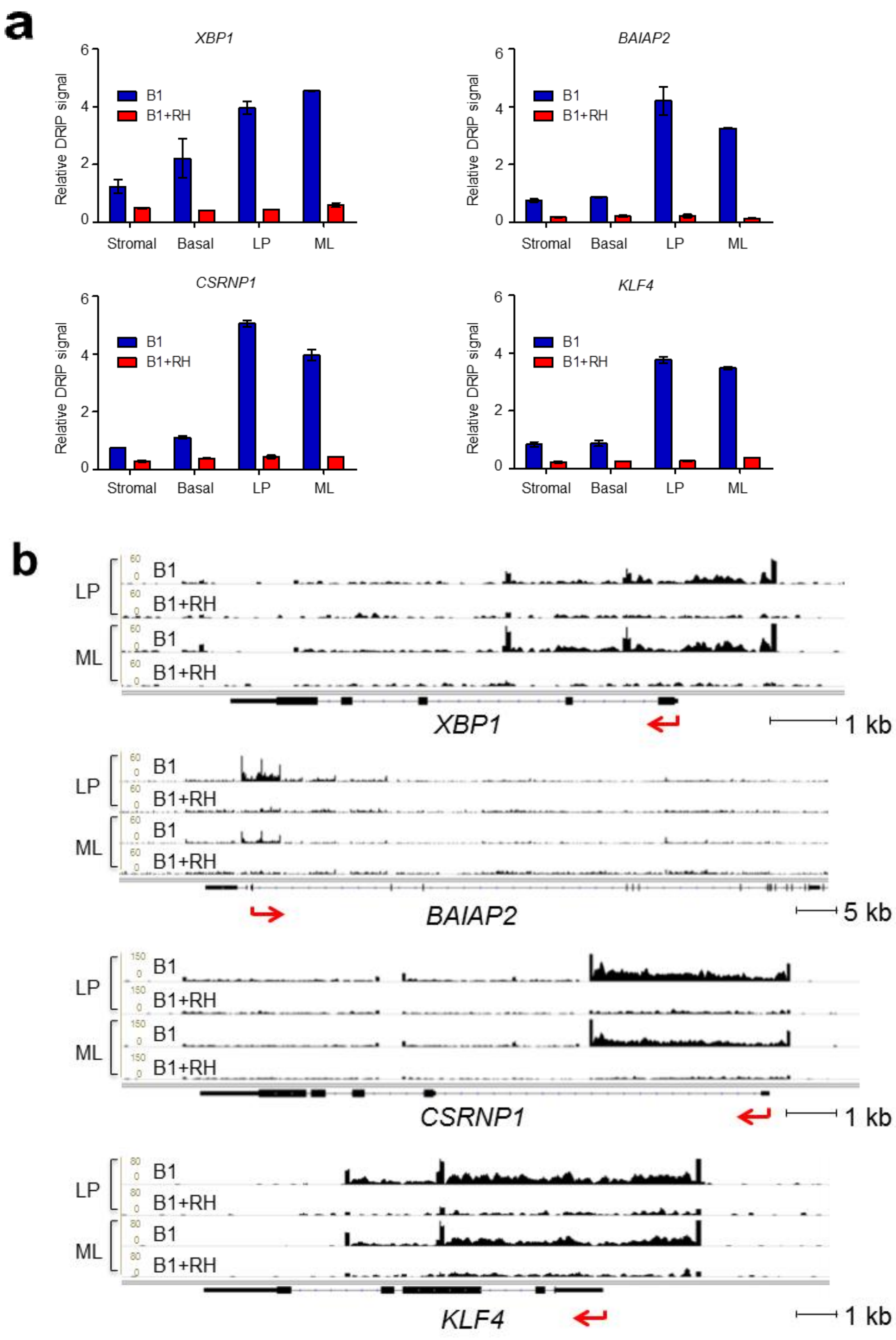

Supplementary Figure 4. RNase H pre-treatment abolishes DRIP and DRIP-seq signals. (**a**) Relative R-loop signal by DRIP at *XBP1*, *BAIAP2*, *CSRNP1* and *KLF4* in a *BRCA1* mutation carrier, with or without pre-treatment of RNase H**.** (**b**) RNase H pre-treatment abolishes DRIP-seq signals. The same DRIP DNA from (a) was subjected to deep sequencing. IGV track view of DRIP-seq density profile centered on *XBP1*, *BAIAP2*, *CSRNP1* and *KLF4*, with or without pre-treatment of RNase H. TSS is marked by a red arrow.

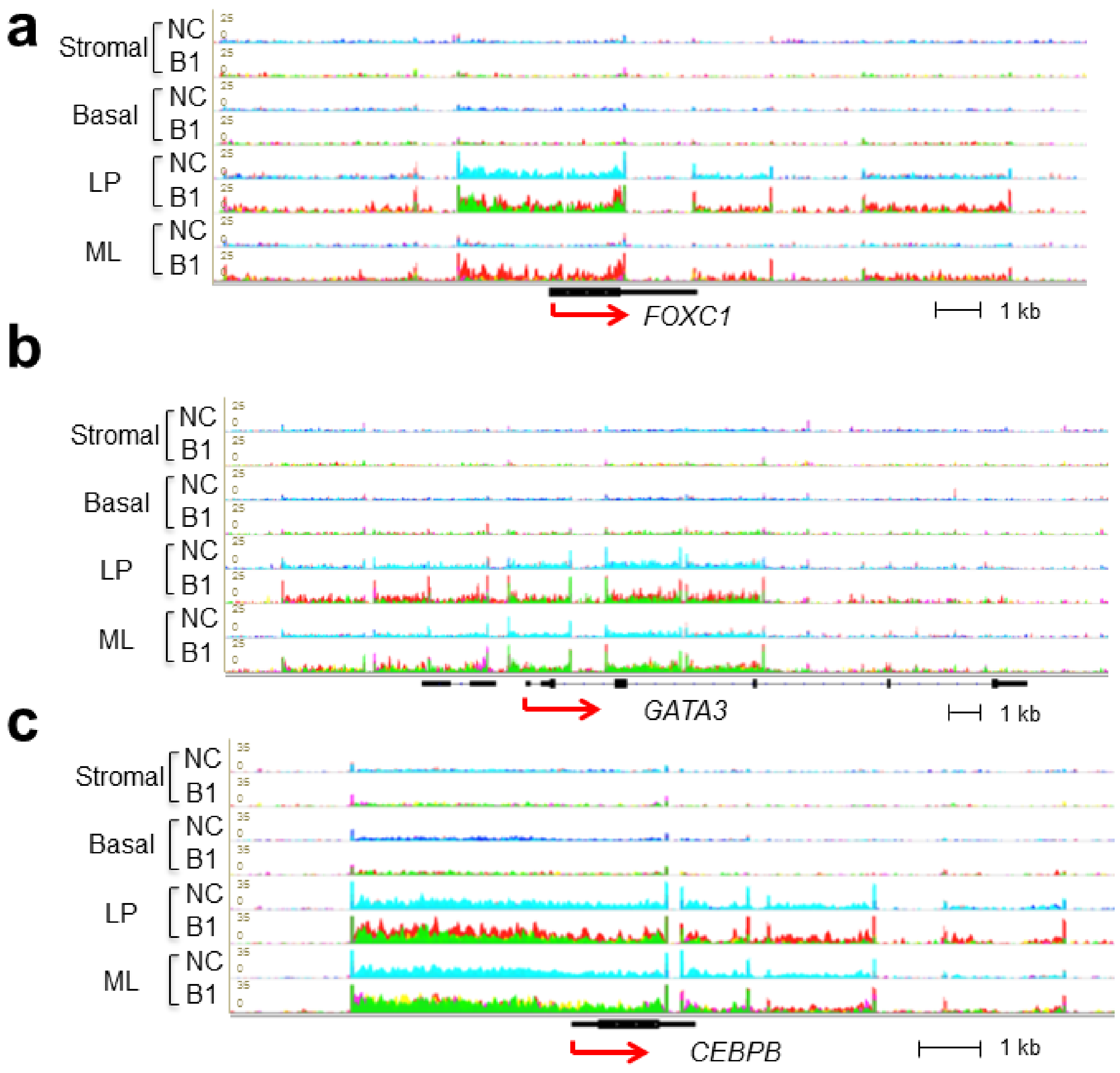

Supplementary Figure 5. IGV for representative luminal genes. Track view of DRIP-seq density profile centered on gene *FOXC1* (**a**), *GATA3* (**b**) and *CEBPB* (**c**). Each track is an overlay of four individual non-carriers or four *BRCA1* mutation carriers indicated by different colors. TSS was marked by red arrow.

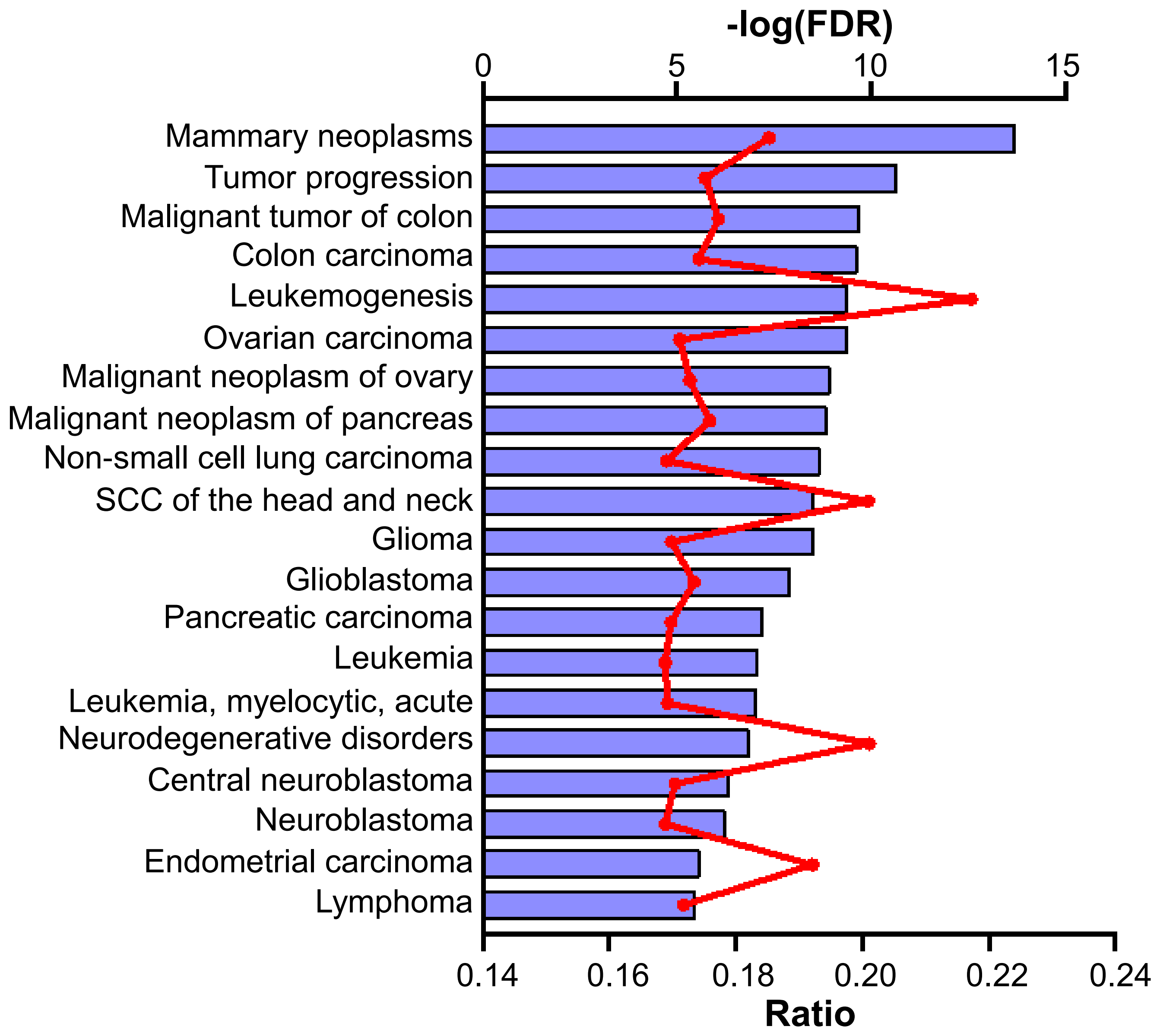

Supplementary Figure 6. Disease association of genes with *BRCA1* mutation-associated R-loops. ToppGene was used to generate disease association.

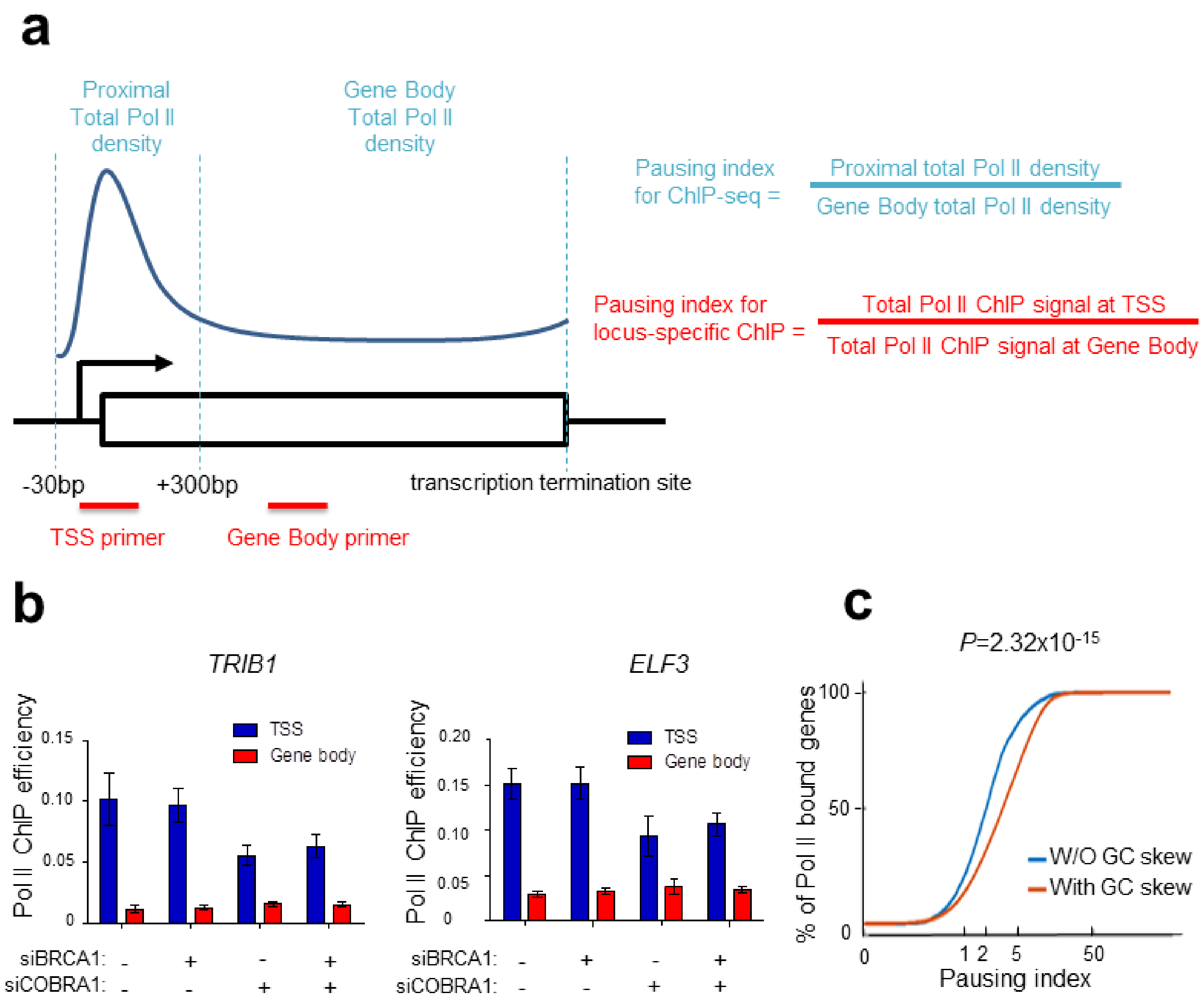

Supplementary Figure 7. Pol II pausing index calculation and correlation between high skewed GC and Pol II pausing. (**a**) Diagram that illustrates calculation of Pol II pausing index for ChIP-seq and locus-specific ChIP. (**b**) Total Pol II signals at TSS and Gene Body at *TRIB1* and *ELF3*. (**c**) Cumulative curve of pausing index for genes with TSS GC skew (red) or without GC skew (blue) in K562 cells. *P* value was calculated using signed rank sum test.

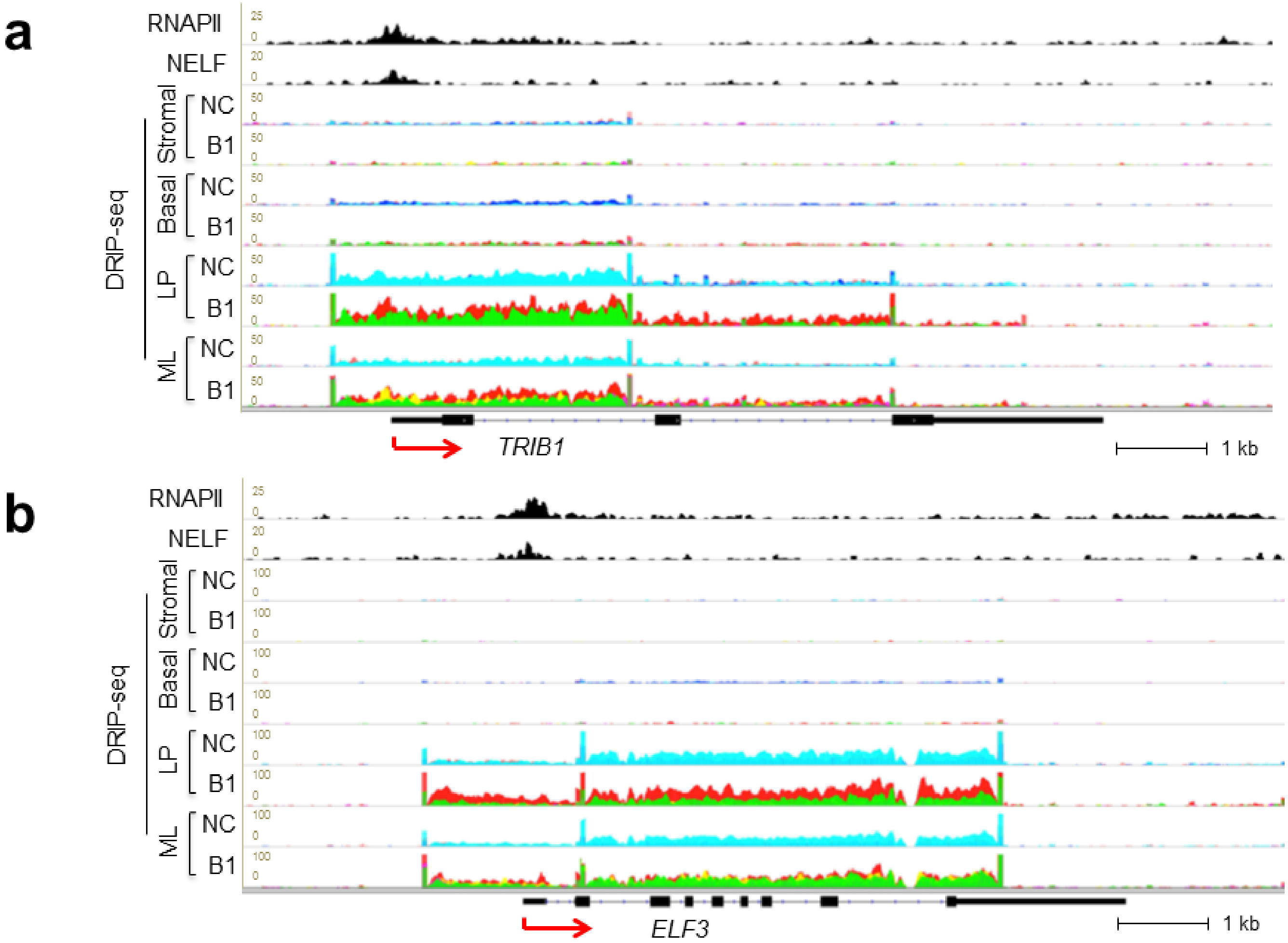

Supplementary Figure 8. IGV of Pol II and NELF ChIP-seq, and DRIP-seq signals at *TRIB1* and *ELF3*. Track view of ChIP-seq and DRIP-seq density profile centered on gene *TRIB1* (**a**) and *ELF3* (**b**). Total Pol II and NELF ChIP-seq experiments were done using human primary breast epithelial cells. Each track is an overlay of four individual non-carriers or four *BRCA1* mutation carriers indicated by different colors. TSS is marked by a red arrow.

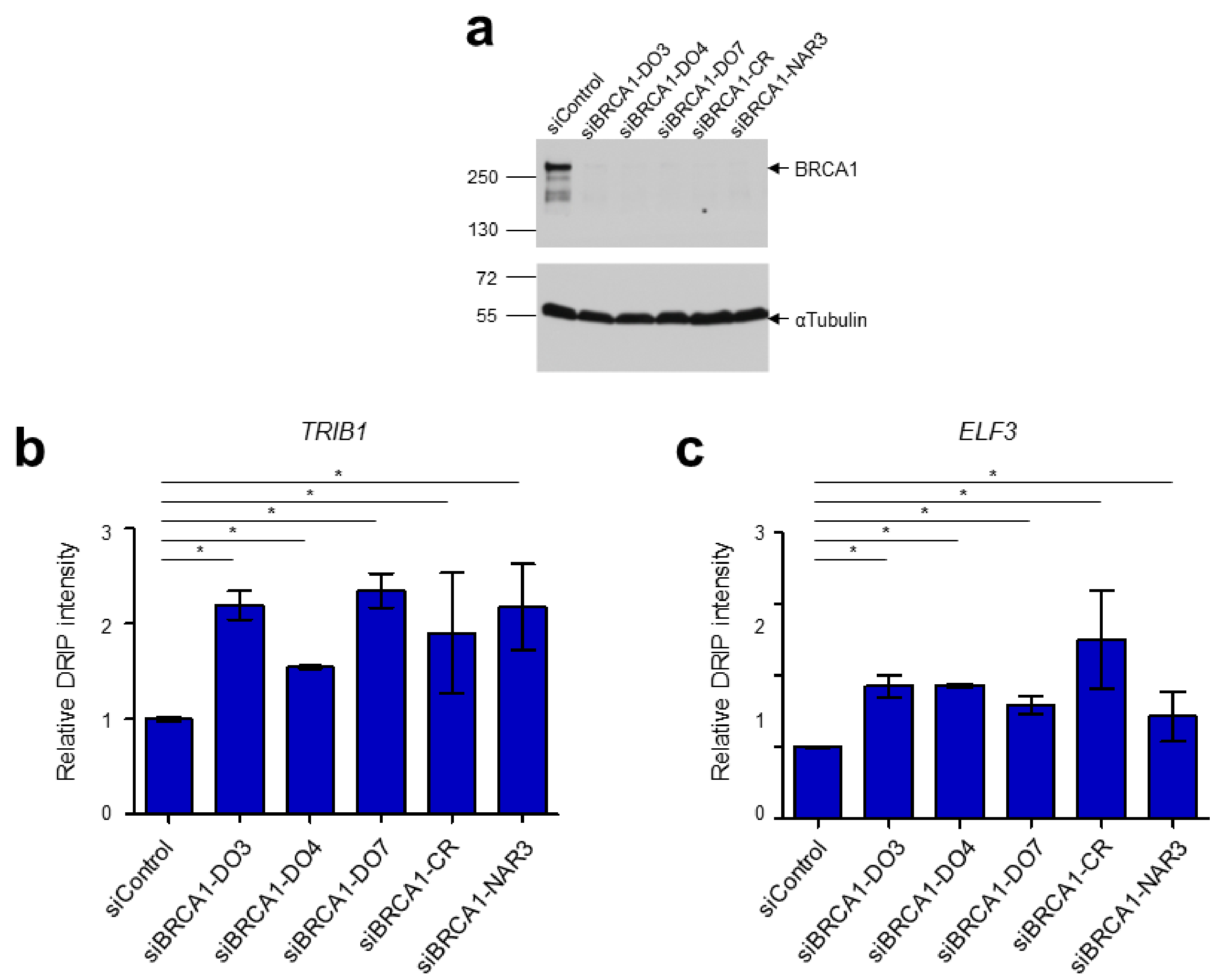

Supplementary Figure 9. Effect of BRCA1 knockdown on R-loop levels at *TRIB1* and *ELF3.* (**a**) Immunoblot confirming BRCA1 knockdown efficiency in T47D cells. α-Tubulin was used as a loading control. (**b,c**) Relative DRIP signal at *TRIB1* (a) and *ELF3* (b) in control and BRCA1 KD with different BRCA1-targeting siRNA oligos in T47D cells. * $P < 0.05$ by two-tailed *t*-test. Error bars represent s.e.m.

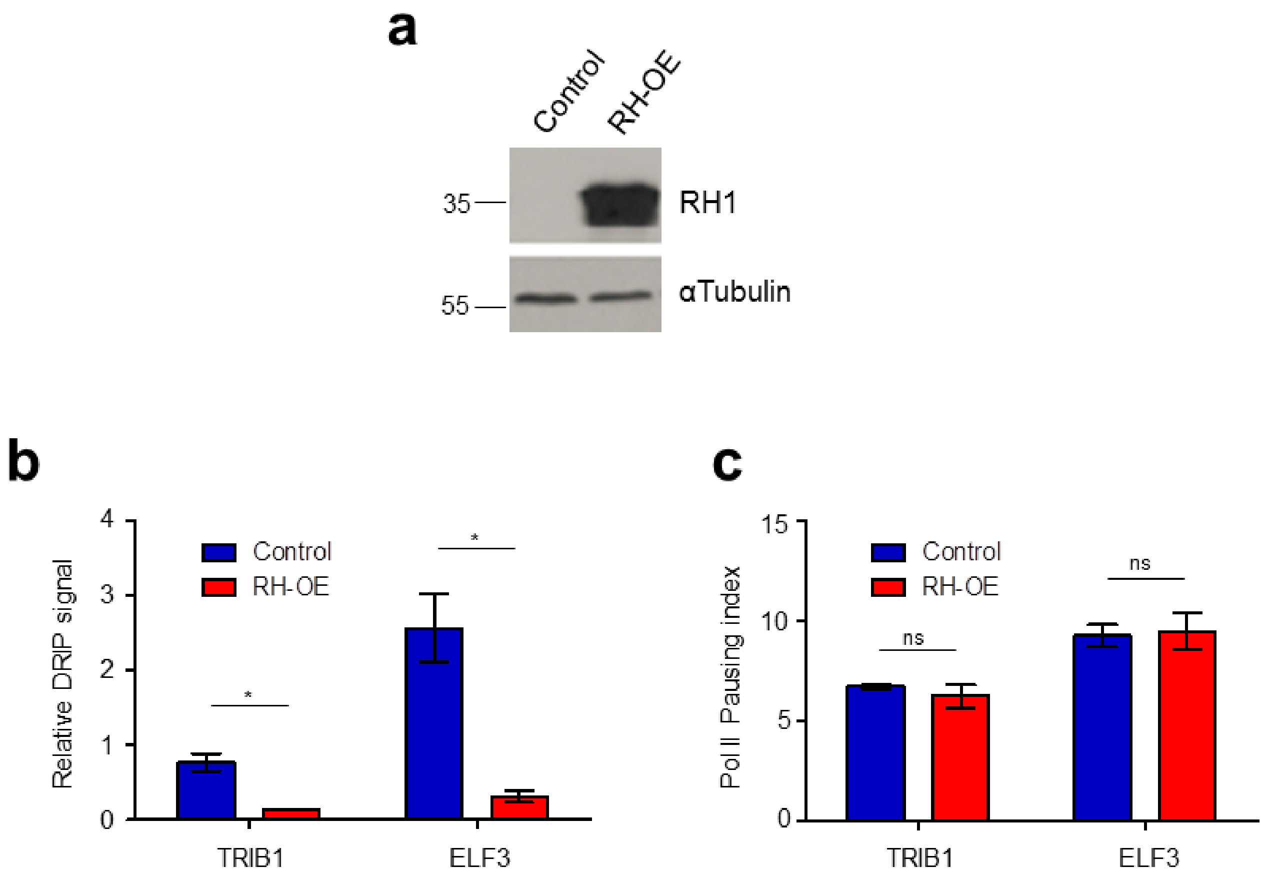

Supplementary Figure 10. Effect of RNase H1 overexpression on Pol II pausing. (**a**) Immunoblot confirming RNase H1 overexpression. α-Tubulin was used as a loading control. (**b**) Relative DRIP signal at *TRIB1* and *ELF3* in control and RNase H1-overexpressed T47D cells. * $P < 0.05$ by two-tailed *t*-test. Error bars represent s.e.m. (**c**) Pol II pausing index at *TRIB1* and *ELF3* loci in control and RNase H1-overexpressed T47D cells. Pol II pausing index is calculated by the ratio of total Pol II signals at TSS over Gene Body. Error bars represent s.e.m. ns: not significant.

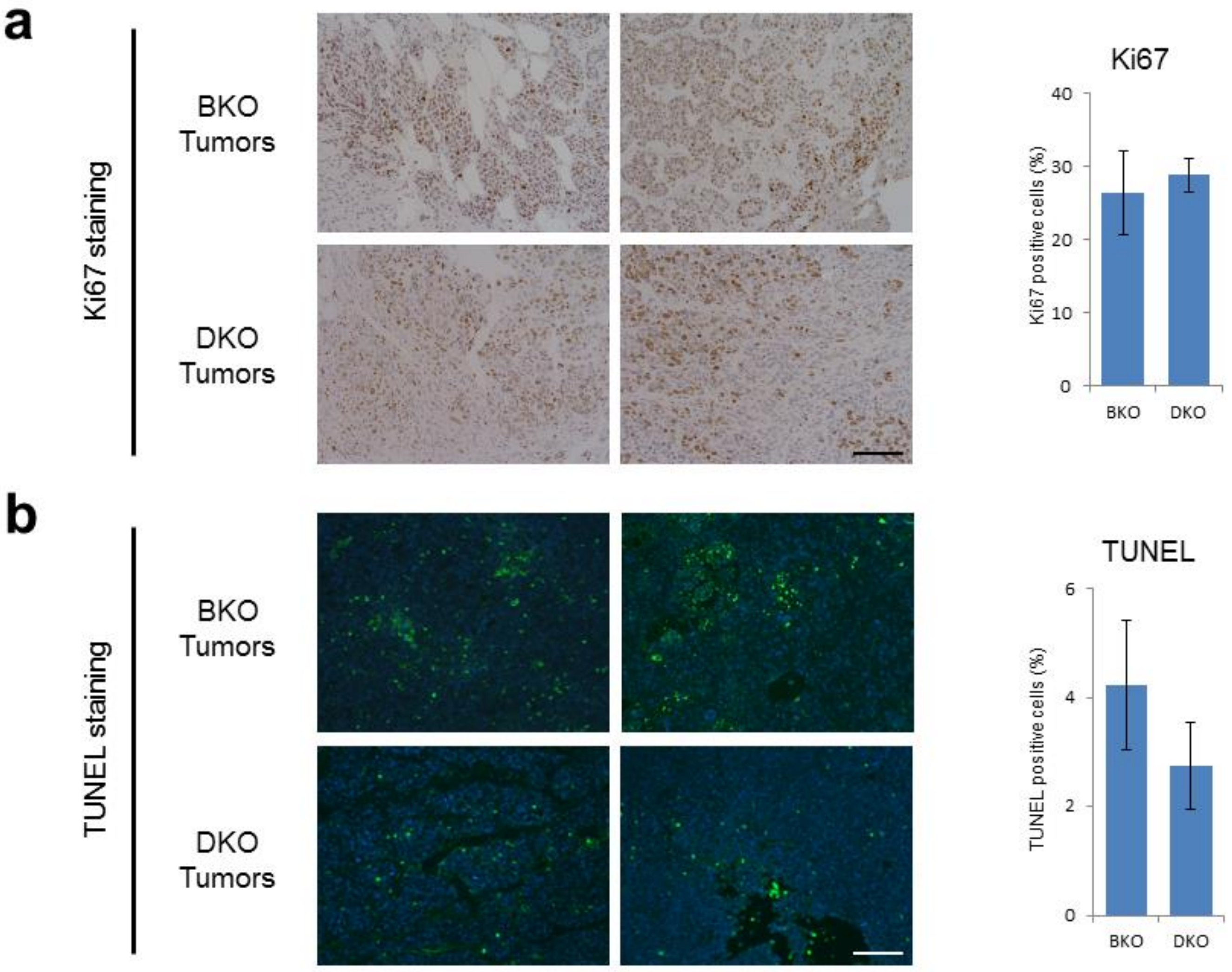

Supplementary Figure 11. Cell proliferation and apoptosis in BKO and DKO tumors. (**a**) Ki67 staining of BKO and DKO tumors, showing representative images from two individual tumors from both groups. Scale bar: 100µM. Bar graph on the right represents quantification of percentage of Ki67-positive cells. BKO tumors: n=7, DKO tumors: n=7. (**b**) TUNEL assay of BKO and DKO tumors, showing representative images from two individual tumor from both groups. Scale bar: 100µM. Bar graph on the right represents quantification of percentage of TUNEL-positive cells. BKO tumors: n=7, DKO tumors: n=7.

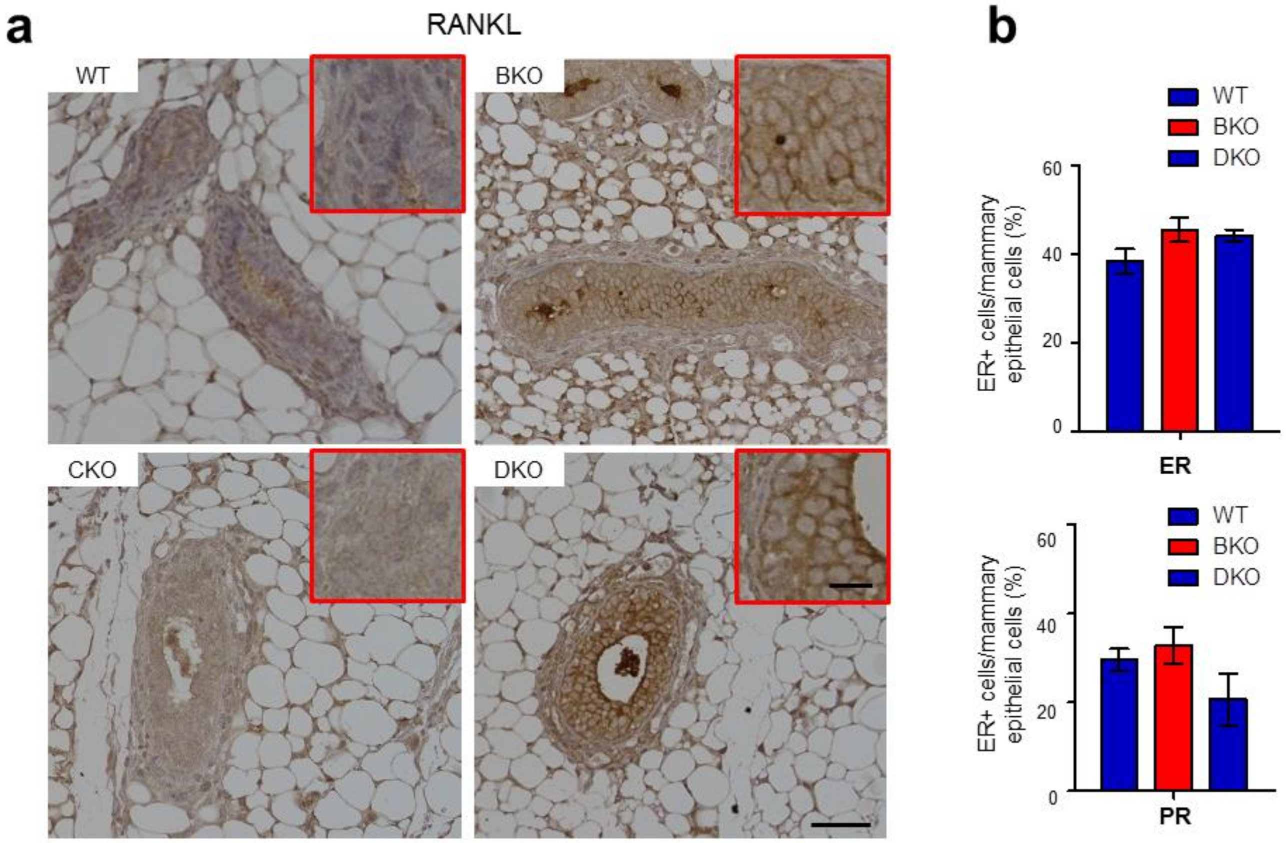

Supplementary Figure 12. Lack of rescue in RANKL overexpression, and lack of changes in ER/PgR+ percentage in DKO mice. (**a**) Low and high (inlet) magnification images of RANKL staining in mammary ducts of 12-week old virgin mice, n=4 in each group. Scale bars: 50 µm and 20 µm (inlet). (**b**) Quantification of ER+ and PgR+ cell percentages in 8-week old virgin mouse mammary epithelia. n=3 in each group.

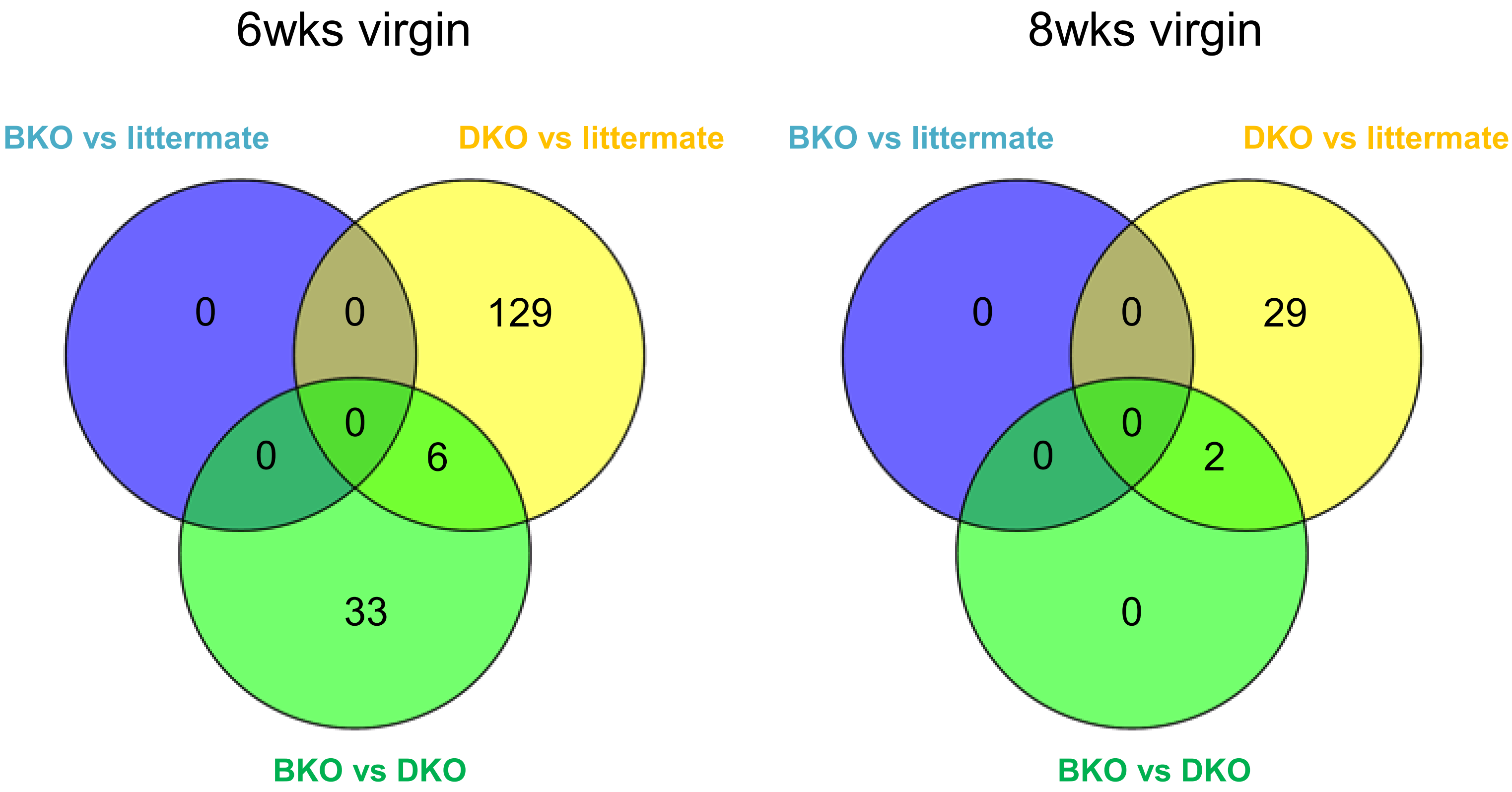

Supplementary Figure 13. Gene expression changes in BKO and DKO mouse mammary epithelia. Venn diagrams depicting the overlap between genes differentially expressed in BKO comparing to control littermates, DKO comparing to control littermates, and BKO comparing to DKO. Microarray was performed using CD24-sorted mammary epithelial cells from either 6 weeks or 8 weeks old virgin mice.

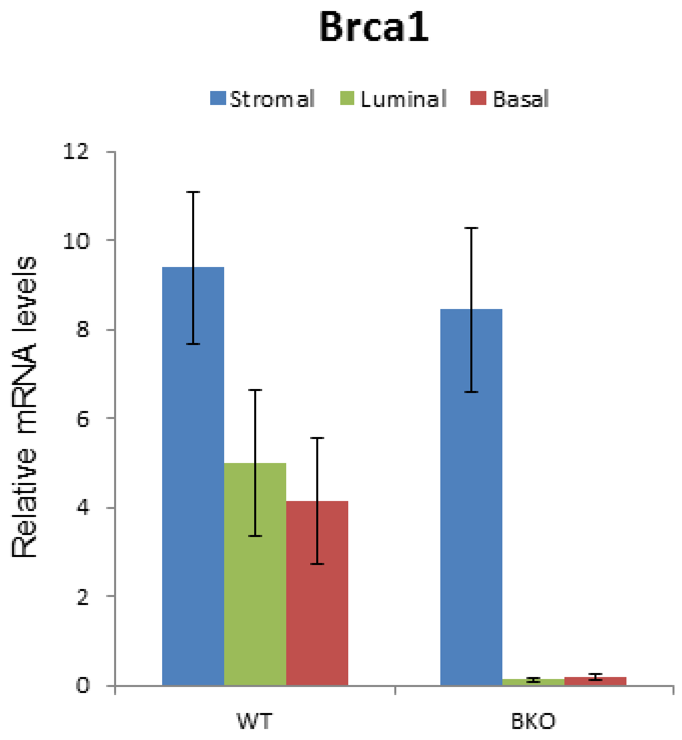

Supplementary Figure 14. Validation of BRCA1 depletion in BKO mammary luminal and basal epithelia. mRNA analysis of *Brca1* expression in sorted stromal ($CD49f^{-}$ $EpCAM^{-}$), luminal ($CD49f^{med}EpCAM^{high}$), and basal ($CD49f^{high}EpCAM^{med}$) cells of 8-week old virgin mice. WT: n=4, BKO: n=5. Error bars represent s.e.m.

Supplementary Table 1. siRNA target sequences

siRNA name	Target sequence
siBRCA1-DO3	GAAGCCAGCTCAAGCAATA
siBRCA1-DO4	GCAGATAGTTCTACCAGTA
siBRCA1-DO7	GAAGGAGCTTTCATCATTC
siBRCA1-CR	AAGGTTTCAAAGCGCCAGTCA
siBRCA1-NAR3	ACCATACAGCTTCATAAATAA
siNELFB	GCGACTTGGCCTTTGGCGA

Supplementary Table 2. Primer sequences for RT-PCR

Name	Forward primer	Reverse primer
VIM	GGAAGCCGAAAACACCCTG	GAGACGCATTGTCAACATCCT
KRT14	CATGAGTGTGG AAGCCGACAT	GCCTCTCAGGGCATTCATCTC
KRT18	ACAATGCCCGCAT CGTTCT	GGATGTCGTTCTCCACAGACT
ESR1	TGCTACGAAGTGGGAATGATGA	ATCTCTCTGGCGCTTGTGTT
ACTB	AGGCACCAGGGCGTGAT	GCCCACATAGGAATTCCTTCTGAC
mBrca1	TCTGCACCACCTCTCCTTGG	AGCAAACAGCCTGGCATAGC
mActb	CGGTTCCGATGCCCTGAGGCTCTT	CGTCACACTTCATGATGGAATTGA

Supplementary Table 3. Primer sequences for DRIP

Name	Forward primer	Reverse primer
TRIB1	TCCAGCCAGCGATTTTCCTT	CGGCGGATCCTGTTTCTAGG
ELF3	GCAAGCGCCATTGACTTCTC	CAAGGGCACAATTGCAGAGG
XBP1	AGGACCGTGGCTATGGAGT	AGTACCTTTGGCCAGGGATTG
BAIAP2	GTATTTACCCGGCAGTCGCT	GCCGCCTACCACAATCAGAA
CSRNP1	CTCTGAGAGTGACGGCGAC	GGAGGGATTGTGTCGTAGGC
KLF4	GAGACCTGTCAGTGGTGGTC	AGCACGTCAGTATGTCGGGT

Supplementary Table 4. Primer sequences for ChIP

Name	Forward primer	Reverse primer
TRIB1-TSS	GCTCGCTCTCATACACGCC	AAAGCGATGAGTCTCCAGCAA
TRIB1-gene body	TCCAGCCAGCGATTTTCCTT	CGGCGGATCCTGTTTCTAGG
ELF3-TSS	TTTAGAGCCGGGTAGGGGAG	CCAGGTAGCGCTGAGGTATC
ELF3-gene body	CCCCAGTTCTGGTCGAAGAC	CGCTTGCGTCGTACTTGTTC
